# Critical dialogue: Interstitial lung abnormalities: an evolving concept

**DOI:** 10.1186/s13244-026-02357-7

**Published:** 2026-08-06

**Authors:** Maurizio Balbi, Roberta Eufrasia Ledda, Rebecca Mura, Venerino Poletti, Anna-Maria Hoffmann-Vold, Helmut Prosch, Nicola Sverzellati

**Affiliations:** 1 Radiology Unit, San Luigi Gonzaga Hospital, Orbassano, Italy; 2 Department of Oncology, University of Turin, Orbassano, Italy; 3 Scienze Radiologiche Unit, University Hospital of Parma, Parma, Italy; 4 Department of Medicine and Surgery, University of Parma, Parma, Italy; 5 Department of Biomedical Imaging and Image-Guided Therapy, Medical University of Vienna, Vienna, Austria; 6 G.B. Morgagni-L. Pierantoni Hospital, Forlì, Italy; 7 Department of Medical and Surgical Sciences (DIMEC), University of Bologna/Forlì-Ravenna Campus, Bologna, Italy; 8 Department of Rheumatology, Oslo University Hospital, Oslo, Norway; 9 Department of Rheumatology, University Hospital Zurich, University of Zurich, Zurich, Switzerland; 10 Christian Doppler Laboratory for Machine Learning Driven Precision Imaging, Department of Biomedical Imaging and Image-guided Therapy, Medical University of Vienna, Vienna, Austria

## Current practice and knowledge

According to the 2020 Fleischner Society position paper, interstitial lung abnormalities (ILAs) refer to incidental findings potentially reflective of unsuspected interstitial lung disease (ILD) on partial or complete CT evaluation of the lungs [1]. The definition of ILAs is imaging-based, whereas separation from ILD relies on ensuing clinical assessment, including physical examination and pulmonary function testing. ILAs are defined as nondependent abnormalities involving ≥ 5% of any lung zone (upper, middle, and lower, demarcated by the levels of the inferior aortic arch and right inferior pulmonary vein) [1]. Individual findings, including ground-glass opacity, reticulation, non-emphysematous cysts, honeycombing, and traction bronchiectasis, are classified into one of three categories: non-subpleural, subpleural non-fibrotic, or subpleural fibrotic ILAs [1]. Fibrotic ILAs require architectural distortion with traction bronchiectasis or honeycombing (or both) and are categorized in accordance with current guidelines as typical, probable, or indeterminate for usual interstitial pneumonia (UIP) [1]. Reticulation alone is not considered fibrotic ILAs because of the lower likelihood of progression and lower mortality in the absence of traction bronchiectasis or bronchiolectasis [1, 2]. Histopathologic correlation remains heterogeneous: subpleural fibrotic ILAs more often correspond to pathologic typical UIP or probable UIP patterns, although several differential diagnoses, especially smoking-related interstitial fibrosis, should be considered [3, 4]. Despite being often underreported [5], ILAs are increasingly recognized given growing evidence of their potential implications, including radiologic progression, decline in pulmonary function, increased respiratory morbidity, and a higher risk of lung cancer and lung cancer–related mortality [2, 5–10].

## Advancements and new developments

The 2025 American Thoracic Society (ATS) clinical statement substantially updates the definition of ILAs [11], building on the 2020 Fleischner Society position paper [1]. ILAs remain nondependent CT abnormalities involving ≥ 5% of a lung zone, but they are no longer required to be incidental [11]. The ATS also proposes operational criteria to distinguish ILAs from ILD based on symptoms, physiology, imaging, and pathology [11]. From a radiological perspective, this requires recognition of CT findings now proposed to define ILD rather than ILAs: a major fibrotic pattern (UIP, probable UIP, fibrotic hypersensitivity pneumonitis, fibrotic nonspecific interstitial pneumonia), a progressive fibrotic component on serial scans, or the presence of fibrotic abnormalities (honeycombing and/or reticulation with traction bronchiectasis) involving ≥ 5% of total lung volume by visual assessment [11]. Notably, the ATS assigns only limited prognostic significance to bronchiolectasis and, unlike traction bronchiectasis, does not incorporate it into the ILD definition [11]. Other suggestions include systematic assessment for ILAs/ILD in lung cancer screening (LCS) participants, patients with connective tissue disease (CTD), and adults aged ≥ 50 years with a first-degree relative with familial pulmonary fibrosis [11].

## Impact on radiology practice

While the ATS’s effort to provide operational guidance is commendable, a few considerations are worth noting. The proposed threshold of ≥ 5% fibrotic abnormalities for ILD may paradoxically widen the gray zone between ILAs and ILD when fibrotic changes are limited. Overt, extensive fibrosis may reasonably be labeled as “disease”—even if incidental—but the same is less straightforward when only scant fibrosis is detected. For example, although Fig. 2 in the ATS statement is labeled as fibrotic ILD [11], many radiologists may hesitate to classify such minimal fibrotic changes as ILD rather than fibrotic ILA. Given that visual quantification of pulmonary fibrosis on CT is heavily influenced by radiologist expertise and subject to observer variability [12], consistent and reliable short-term application of this threshold in routine care may be difficult. Quantitative CT, in its various forms, could support radiologists in this task, although its translation into routine care remains limited despite more than a decade of research interest [13, 14]. In this context, artificial intelligence has shown potential to facilitate detection of subtle early imaging features, while technical advances in CT, including improved spatial resolution of photon-counting detector CT, may contribute to earlier identification of ILAs and more precise depiction of fine fibrotic abnormalities [15].

Another practical concern is the proposed distinction between bronchiolectasis (defined by the ATS as dilation of bronchioles within the central portions of the secondary pulmonary lobule) and bronchiectasis, as well as the exclusion of bronchiolectasis from the fibrotic burden [11, 16]. In reporting practice, this distinction can be cumbersome as bronchiectasis and bronchiolectasis lie along the same continuum of airway remodeling. Since both findings are associated with clinically relevant outcomes [17–19] and contribute to the definition of UIP and probable UIP patterns [20], the practical value of the newly proposed approach remains debatable.

The pretest probability of encountering ILAs or ILD varies substantially among LCS participants, patients with CTD, and first-degree relatives of patients with familial pulmonary fibrosis. This raises the question of whether applying the same criteria across these settings is appropriate, as similar imaging findings may carry different clinical meanings depending on the clinical context. For instance, bilateral subpleural reticulation associated with scant traction bronchiolectasis in a 40-year-old woman with a previous diagnosis of systemic sclerosis can now be classified as subpleural fibrotic ILA if symptoms and pulmonary function abnormalities are absent. Since these findings would arguably reflect pulmonary involvement related to the underlying CTD unless proven otherwise [21]—and thus constitute disease by definition—the term ILA, in this scenario, may serve more as an indirect surrogate for disease severity than as a threshold of clinical significance. In contrast, in a 70-year-old man undergoing LCS, the same abnormalities may still be classified as subpleural fibrotic ILA yet may help stratify risk of actionable disease, such as idiopathic pulmonary fibrosis, vs smoking- or age-related changes.

Although the suggestion of screening high-risk populations for ILAs/ILD may create opportunities for early disease identification and intervention, it may also place additional strain on healthcare systems, as acknowledged by the ATS [11]. This concern is primarily related to the inclusion of high-prevalence CTDs, notably rheumatoid arthritis, and to the expanding implementation of LCS across European and non-European countries. While costs and burden may be mitigated in LCS because imaging is already performed for lung cancer detection, the balance between surveillance and early pulmonology referral for ILAs in this setting remains to be defined, potentially resulting in additional, and sometimes unwarranted, examinations [22].

## Data Availability

Not applicable.

## Abbreviations

ATS: American Thoracic Society
CTD: Connective tissue disease
ILA: Interstitial lung abnormality
ILD: Interstitial lung disease
LCS: Lung cancer screening
UIP: Usual interstitial pneumonia
